# Supplementary material for: Exploratory and confirmatory factor analysis of the new region-generic version of Fremantle Body Awareness—General Questionnaire
Source: PLoS One. 2023 Mar 22;18(3):e0282957. doi: 10.1371/journal.pone.0282957 (PMC10032497; doi:10.1371/journal.pone.0282957)
Supplement: Attachment — Submitted filename: Response to Reviewers.docx [file pone.0282957.s002.docx]

We would like to thank the reviewers for the valuable feedback and comments.

**Response:** As reviewer #1 pointed out there are indeed several additional things we could have done with the data, though they either do not add substantive meaning to the results or interpretation therefore or add extra weight to an already dense paper. Per the comparative fit indicators, those behave as one would expect based on the other indicators we’ve described (e.g., AIC goes from 3273.96 for the uncorrelated 7-item model, to 2755.72 for the 6-item model with residuals of items 1 and 5 correlated). If we included such an indicator, we’d need to also describe it in the Analysis and interpret it in the Discussion, but it does not change the final story so in the interest of parsimony we, respectfully, do not agree with inclusion of those additional metrics unless it becomes a barrier to moving the paper forward. Reviewer also pointed out that it is to the Editor’s discretion. Though again, this is already a dense paper describing a number of analyses. We would like to hold back these additional components for a subsequent manuscript, and for that matter we’ve also got plans to explore the tool through Rasch or IRT for even further detail. Something needs to be held back, and we propose that the analyses described herein are enough to endorse utility of the tool, with the acknowledgment that PROMs always have room for further improvement.
